# Malignant Growths

**Published:** 1894-06-30

**Authors:** 


### MALIGNANT GROWTHS.

**Rodent Ulcer.**—The subject of rodent ulcer has lately been fully discussed at the Pathological Society.[1] It was shown that it often begins considerably before the period of old age, though it is generally considered as a senile disease. In 66 cases Bowlby found it 10 times between the ages of 35 and 40, 16 times between 40 and 50, 15 times between 50 and 60, and only 9 times after 60. In most of Bowlby's cases the disease began as a wart. In several there was no ulceration for years. An equal number had raised, thickened, and more or less indurated margins, and flat and sharply-cut edges. Both forms presented the same histological characters. It was extremely rare except on or close to the face. Bowlby believes it is not in any way an epidermal growth, but what structure in the derma it arises in he does not seem certain.

Mr. Hutchinson quite agreed with Mr. Bowlby that rodent ulcer was not confined to old age. Mr. Paul and Dr. Kanthack were decidedly of opinion that the growth started in the sebaceous glands. Mr. Paul showed specimens of small rodent ulcers in which, whilst the epidermis, hair follicles, and sweat glands were normal, the sebaceous glands were "entirely replaced by new growth." Dr. Norman Walker had, however, observed atrophy of the sebaceous glands as rodent ulcer growth invaded the tissues. Dr. Kanthack thought he had actually seen the epithelial masses arising in connection with a sebaceous gland, and the arrangement of these masses suggested such an origin. Dr. Rupert Boyce believed in their origin in sebaceous glands. Other speakers were inclined to trace their development to hair follicles. During the discussion Dr. Kanthack referred to the fact that ingrowth of epithelium might occur with any form of irritation, and that he had seen it in laryngeal catarrh and in the tongue before leucoplakia developed.

**The Relation Between Arsenic and Epithelioma.**—Mr. H. Arbuthnot Lane, at the Clinical Society,[2] read an account of multiple epitheliomatous formations in a very persistent condition of psoriasis treated by arsenic. There had been during the last few years eleven epitheliomatous growths. Four were on the forearm and seven on the scrotum and perineum. They did not occur in patches of psoriasis, but in healthy skin. The glands do not appear to have suffered, and only one growth was a recurrence. Mr. Hutchinson had seen a case in which "little corny indurations" formed in the palms and soles, which became epitheliomatous. He considered these corneous growths a warning to leave off the arsenic. Mr. Hulke had been to Devonshire to see how the workers in arsenic mines were affected. He found them broken down in health, but in no instance had epithelioma arisen in connection with their exposure to arsenic.

**Protozoa and Cancer.**—What is the present position of our knowledge of protozoa and carcinoma is a question very ably discussed by Dr. Adler.[3] He has been engaged in very laborious investigations himself with the tissues hardened in all sorts of ways, with the help of many fellow workers, and has consulted no less than 84 communications on the subject, a reference to which is given, and amongst them we see many of last year's date, so that he has given us very recent views. He points out the widely divergent views held by numerous English, American, and Continental pathologists on the subject, and considers himself that no case has been satisfactorily established for the protozoa, either as constantly existing in cancer, or still less as casual agents. He refers to the frequent mistakes made in regarding as protozoa cell and nuclear changes and inclusion, and points out that there is no specific stain. The conditions under which we may believe in the presence of protozoa in cancer he summarised as follows: "We are justified in making a diagnosis of protozoan parasite in cancer only when its morphological attributes and its reaction towards stains are such as to preclude all possibility of being explained by any of the numerous kinds of cell metamorphosis or other irregularities of cell life hinted at above, and when in addition thereto a sufficiently well marked and well established developmental cycle can be demonstrated." The failure in attempts to obtain cultivations of the protozoa is recalled to our memory, and we are told that, according to Koch's formula, we must find a specific micro-organism constantly occurring in every case of carcinoma, and in such distribution and topographical arrangement as would suffice to explain the anatomical facts: pure cultures must be obtained, and inoculation of these must produce the disease. These conditions are all wanting. A very important statement of Dr. Adler's with regard to secondary growths is made. He has examined the very earliest metastatic growths in the glands, when they only consisted of a few epethelial cells amongst the lympatic tissue, where certainly one ought to find the protozoa-like bodies, if they are protozoa, and has never found any. There is also another difficulty in accepting the protozoan theory of the cause of cancer. If the bodies were the cause, surely they would by their presence amongst the cells of the glands or liver, where they lodge as emboli and set up secondary growth, stimulate a proliferation of the tissue of the organ, but they do not, the secondary growth exactly resembles the parent growth.

**The Alleged Increase of Cancer.**—A valuable paper on the mortality from cancer has lately been contributed by Mr. George King and Dr. Arthur Newsholme to the Royal Society, and an abstract of it, at some length, appears in the *Lancet*.[4] Mr. King is a skilled actuary, and has gone most carefully into the statistics. They think that the supposed increase in cancer is only apparent, and is due to improvement in diagnosis and greater care in giving certificates of death. More accurate registration under more definite headings is needed before the matter can be definitely settled. At Frankfort-on-the-Main, where unique statistics are kept, the figures show that cancer of "accessible" parts in the female did not increase, but did so to a marked degree in the inaccessible, which looks as if the apparent increase was due to more careful observation.

**Medullary Sarcoma of Sternum with Metastases in Lymphatic Glands.**—The case is described in the "Johns-Hopkins' Hospital Bulletin."[5] The disease first showed itself in the form of a swelling in the suprasternal notch. The cervical and axillary glands enlarged, and nodules were found beneath the skin of the upper part of the thorax. The sternal growth rapidly made its appearance at the side of the bone, and intrathoracic pressure symptoms developed. Post-mortem, the disease was found to have infiltrated the sternum and lung, and separate nodules of growth were found in the lungs. The microscopic characters of the sternal, lung, and lymphatic gland growth was that of round celled sarcoma, myeloid cells being present.

**Congenital Sarcomata of Kidneys.**—Mr. Paul has lately written on this subject in the *Liverpool Medico-Chirurgical Journal*.[6] He considers that they are primarily extra-renal, though usually intra-capsular, and they distend or surround the kidney in preference to infiltrating it. They do not cause urinary symptoms as a rule. In about half the cases they are bilateral. All recur after removal. The very interesting feature in their histology is the fact that they often contain striped muscular fibres and embryonic renal tissue.

**Transformation of Innocent into Malignant Growth in the Uterus.**—An undoubted example of this condition was exhibited by Dr. Lee Dickinson at a meeting of the Pathological Society.[7] A typical fibro-myoma of the uterus was found post-mortem to have formed in one part a sprouting hæmorrhagic growth. A portion of the growth was very soft, and had broken down into the uterus, and also into the peritoneal cavity. The sarcomatous portion consisted of short spindle cells with enormous nuclei.

Mr. Doran considered that not only could a fibromyoma change into sarcoma, but that carcinoma might arise in such a growth, and stated that Schottländer had quite recently proved that glandular and epithelial growths might be found deeply situated in myomatous tumours, from which such carcinomatous transformations might arise.

[1] Lancet, Feb. 24, 1894, p. 477, and March 18, p. 603; also Med. Week., March 9, 1894, p. 113. [2] Lancet, Feb. 17, 1894, p. 408. [3] Amer. Jour. Med. Sciences, Jan., 1894, p. 63. [4] Lancet, Feb. 3, 1894. [5] Ibid., Nov., 1893. [6] Ibid., No. 26, Jan., 1894, p. 102. [7] Ibid., Jan. 6, 1894, p. 22.

